# Selective C–C Coupling Reaction of Dimethylphenol to Tetramethyldiphenoquinone Using Molecular Oxygen Catalyzed by Cu Complexes Immobilized in Nanospaces of Structurally-Ordered Materials

**DOI:** 10.3390/molecules20023089

**Published:** 2015-02-12

**Authors:** Zen Maeno, Takato Mitsudome, Tomoo Mizugaki, Koichiro Jitsukawa, Kiyotomi Kaneda

**Affiliations:** 1 Department of Materials Engineering Science, Graduate School of Engineering Science, Osaka University, 1-3, Machikaneyama, Toyonaka, Osaka 560-8531, Japan; E-Mails: maeno@cheng.es.osaka-u.ac.jp (Z.M.); mitsudom@cheng.es.osaka-u.ac.jp (T.M.); mizugaki@cheng.es.osaka-u.ac.jp (T.M.); jitkk@cheng.es.osaka-u.ac.jp (K.J.); 2 Research Center for Solar Energy Chemistry, Osaka University, 1-3, Machikaneyama, Toyonaka, Osaka 560-8531, Japan

**Keywords:** immobilized copper catalyst, poly(propylene imine) dendrimer, magadiite, oxidative coupling, dimethylphenol, diphenoquinone

## Abstract

Two high-performance Cu catalysts were successfully developed by immobilization of Cu ions in the nanospaces of poly(propylene imine) (PPI) dendrimer and magadiite for the selective C–C coupling of 2,6-dimethylphenol (DMP) to 3,3',5,5'-tetramethyldiphenoquinone (DPQ) with O_2_ as a green oxidant. The PPI dendrimer encapsulated Cu ions in the internal nanovoids to form adjacent Cu species, which exhibited significantly high catalytic activity for the regioselective coupling reaction of DMP compared to previously reported enzyme and metal complex catalysts. The magadiite-immobilized Cu complex acted as a selective heterogeneous catalyst for the oxidative C–C coupling of DMP to DPQ. This heterogeneous catalyst was recoverable from the reaction mixture by simple filtration, reusable without loss of efficiency, and applicable to a continuous flow reactor system. Detailed characterization using ultraviolet-visible (UV-vis), Fourier transform infrared (FTIR), electronic spin resonance (ESR), and X-ray absorption fine structure (XAFS) spectroscopies and the reaction mechanism investigation revealed that the high catalytic performances of these Cu catalysts were ascribed to the adjacent Cu species generated within the nanospaces of the PPI dendrimer and magadiite.

## 1. Introduction

The oxidative C–C coupling reaction of 2,6-dimethylphenol (DMP) has attracted interest as a synthetic method for 3,3',5,5'-tetramethyldiphenoquinone (DPQ), which is a useful building block of aryl epoxy resins [1] and acceptor-doped polymers [2], and an activator for redistribution of poly(2,6-dimethyl phenylene ether) (PPE) [3]. Traditionally, the oxidative C–C coupling of DMP to DPQ has been carried out using excess amounts of stoichiometric metal oxidants such as Mn(OAc)_3_ [4], FeCl_3_·6H_2_O [5], Co(OAc)_3_ [6], and Ph_3_Bi(OAc)_2_ [7]. However, these methods suffer from the production of a large quantity of harmful metal wastes.

With respect to environmental impact and atom efficiency, one of the most promising methods is the catalytic oxidative coupling of DMP to DPQ with O_2_ as an oxidant because only H_2_O is formed as the co-product. In this context, various transition metal catalysts have been applied to the aerobic coupling reaction of DMP. However, the control of the selectivity for C–C coupling has been difficult, resulting in the occurrence of side reactions such as C–O coupling of DMP to PPE and oxidation of DMP to dimethylquinone (DMQ) [8,9,10,11,12,13,14,15]. Up to now, few successful catalyst systems for selective C–C coupling of DMP to DPQ using O_2_ have been reported. Bhalerao *et al.* reported the first demonstration of the regioselective coupling of DMP to DPQ by using enzyme *mushroom* *tyrosinase* in 1990. *Mushroom* *tyrosinase*, which has a dinuclear copper species as an active center, promotes the selective C–C coupling in a phosphate buffer solution at room temperature [16]. Since this report, several homogeneous catalysts have been developed [17,18,19]. For example, Mukherjee *et al.* reported a biomimetic copper catalyst [(α,α'-bis[(*N*-methyl-2-pyridyl)ethylamino]-2-fluoro-*m*-xylene)Cu_2_(MeCN)_2_][ClO_4_]_2_ [17], and Liu *et al.* developed a binuclear copper catalyst Cu_2_(bpnp)(μ-OH)(TFA)_3_ (bpnp = bis(2-pyridyl)-1,8-naphthyridine) [18]. The heteropolyanion H_5_PV_2_Mo_10_O_40_ was also found to catalyze the C–C coupling of DMP to DPQ by Lissel *et al.* [19]. However, these catalyst systems have not yet addressed problems with low activity, and/or difficulty in catalyst separation and reuse. Consequently, the development of efficient and reusable catalytic systems for the selective C–C coupling of DMP to DPQ is still in high demand.

A dendrimer is a spherical macromolecule having a regularly branched framework from a core to termini. One of the unique features, different from linear and branched polymers, is an internal nanovoid confined by a regularly branched structure, which enables accommodation of various organic/inorganic molecules within the void space [20,21]. The internal nanovoid serves as a platform for the synthesis of unique metal species including metal nanoclusters [22,23] and the encapsulated metal species are applicable as catalysts for organic reactions [24]. Our research group has synthesized various dendrimers encapsulating mononuclear metal complexes [25], metal nanoparticles [26], and subnano metal clusters [27,28] and explored their unique catalyses for selective molecular transformations. In the course of our studies on these dendrimer-encapsulated metal catalysts, a poly(propylene imine) (PPI) dendrimer-immobilized Cu complex catalyst was developed for the regioselective coupling reaction of DMP to DPQ with O_2_ as an oxidant [29]. The PPI dendrimer encapsulated Cu ions to generate unique adjacent Cu species within the nanovoids, exhibiting a higher catalytic activity compared to previously reported catalysts.

This article describes a comprehensive study of the development of the above dendrimer-encapsulated Cu complex catalyst. Furthermore, in order to realize a more efficient catalyst system with easy handling and high durability, we newly devise a Cu^2+^-exchanged magadiite (Cu^2+^-magadiite) as a heterogeneous catalyst for the selective oxidative coupling reaction of DMP to DPQ. Cu^2+^-magadiite is easily separable from the reaction mixture by filtration and reusable without loss of activity or selectivity. Cu^2+^-magadiite also can be applied to a continuous flow reactor system, achieving a multigram-scale synthesis of DPQ. The detailed characterizations using UV-vis, FTIR, XRD, XAFS, and ESR measurements of these dendrimer-encapsulated and magadiite-immobilized Cu catalysts reveal that the adjacent Cu species generated within the nanospaces of dendrimer and magadiite play a key role in the efficient oxidative coupling of DMP to DPQ.

## 2. Results

### 2.1. Preparation of Immobilized Cu Catalysts

#### 2.1.1. PPI Dendrimer-Encapsulated Cu Catalyst

A PPI dendrimer possesses tertiary amino groups at branching points and primary amino groups at termini, which act as metal coordination sites [30] and base sites [31]. The modification of the terminal amino groups with bulky triethoxybenzamide groups creates confined internal nanovoids within the PPI dendrimer, which can encapsulate metal ions. The triethoxybenzamide-terminated fourth-generation PPI dendrimer (G_4_-TEBA) was synthesized through the reaction between NH_2_-terminated G_4_-PPI dendrimer (G_4_-NH_2_) with 3,4,5-triethoxybenzoyl chloride [26]. Next, a series of G_4_-TEBA-encapsulated Cu^2+^ complexes G_4_-Cu^2+^_n_ (n denotes the number of Cu^2+^ ions in the G_4_-TEBA, n = 2, 8, 12, 16, and 24) were prepared by stirring a mixture of CuCl_2_ and G_4_-TEBA (CuCl_2_ to G_4_-TEBA molar ratio was n:1) in CH_3_CN/CHCl_3_ (1:2 v/v) under an Ar atmosphere [32]. The resulting solution was evaporated to afford G_4_-Cu^2+^_n_ as brown waxy solids.

#### 2.1.2. Magadiite-Immobilized Cu Catalyst

Magadiite (Na_2_Si_14_O_29_·nH_2_O) is a layered silicate consisting of negatively charged silicate layers and Na^+^ cations in the interlayer nanospace, and has characteristics such as easy availability by a simple hydrothermal procedure [33] and high cation-exchange capacity (180 meq/100 g) [34]. The accessibility and cation-exchange ability of magadiite have led to several applications, for example, adsorbents of metal species [35] and building units of photofunctional materials [36].

Magadiite was synthesized by a hydrothermal reaction of SiO_2_, NaOH, and H_2_O [33]. The magadiite-immobilized Cu complex was prepared by a cation exchange reaction (see Experimental Section). In brief, magadiite was added to the methanol solution containing Cu(ClO_4_)_2_·6H_2_O and *N*,*N*,*N*',*N*'-tetramethylethylenediamine (TMEDA) (molar ratio of TMEDA to copper was 1:1), and then the resulting mixture was stirred at 313 K. The obtained solid was filtered, washed, and dried, affording Cu^2+^-magadiite as a light blue powder.

### 2.2. Characterization of Immobilized Cu Catalysts

#### 2.2.1. PPI Dendrimer-Encapsulated Cu Catalyst

A series of G_4_-Cu^2+^_n_ was characterized by UV-vis, FTIR, ESR, and XAFS analyses. G_4_-Cu^2+^_n_ exhibited a d-d transition band at 819 nm, whereas Cu^2+^ ions in the absence of G_4_-TEBA showed a broad absorption peak centered at 860 nm [32]. The absorption intensity at 819 nm increased upon increasing the amount of Cu ions (ε ≈ 130 M^−1^cm^−1^), indicating the encapsulation of Cu^2+^ ions to G_4_-TEBA [32]. Based on the titration of G_4_-TEBA with CuCl_2_ in CHCl_3_/CH_3_CN at 819 nm, the maximum number of Cu^2+^ ions encapsulated within a G_4_-TEBA was estimated to be 30 [30,32]. Similar phenomena and the determination of the maximum number of Cu^2+^ ions in a dendrimer based on spectrophotometric titration were reported by Crooks *et al.*, where the PPI dendrimer encapsulated Cu^2+^ ions in CHCl_3_/MeOH solvent [30]. Additionally, the absorption band attributed to the LMCT of N- and Cl-Cu was observed at approximately 300 nm [32,37,38].

FTIR analysis was carried out to gain structural information on the encapsulated Cu species of G_4_-Cu^2+^_n_ [32]. It was reported that the treatment of Cu mono- and di-nuclear complexes with NaN_3_ gave terminal Cu azide complexes and bridged Cu azide complexes, exhibiting a single peak around 2040 cm^−1^ and two peaks around 2070 cm^−1^ derived from the N_3_ asymmetric stretch (ν_as_(N_3_)), respectively [39]. When treating G_4_-Cu^2+^_n_ (n = 2 or 8) with NaN_3_, the FTIR spectrum exhibited a peak around 2048 cm^−1^ derived from the ν_as_(N_3_) of the terminal mononuclear Cu azide complex. As n increased above 12 (n = 12, 16, and 24), the FTIR spectra of the Cu-azide complexes exhibited two peaks around 2055 and 2080 cm^−1^. Both peaks were attributed to the ν_as_(N_3_) of μ-1,1-binuclear Cu azide complex. The above FTIR measurements indicated that adjacent Cu species were generated within G_4_-Cu^2+^_n_ (n ≥ 12). As a control experiment, we synthesized a branched polyethyleneimine modified with TEBA groups (PEI-TEBA) as an irregularly branched polyamine [32], and prepared the NaN_3_-treated PEI-Cu^2+^ complex (the molar ratio of tertiary amino groups to Cu^2+^ was adjusted to that in G_4_-Cu^2+^_12_). The FTIR spectrum showed a peak corresponding to the ν_as_(N_3_) of terminal mononuclear Cu azide complex at 2048 cm^−1^, indicating the formation of monomeric Cu^2+^ species in PEI-Cu^2+^. Consequently, the internal nanovoid confined by the regularly branched structure of the dendrimer is essential to form adjacent Cu species.

**Figure 1:**
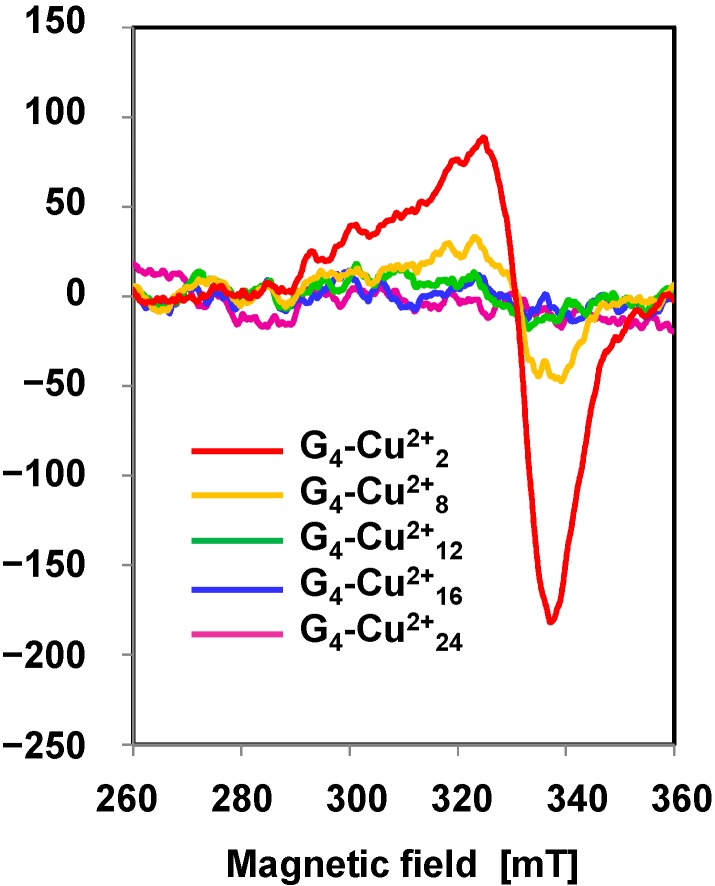
ESR spectra of G_4_-Cu^2+^_n_ (n = 2, 8, 12, 16, and 24) in CHCl_3_ recorded with the following parameters: temperature: 298 K; power: 10.0 mW; modulation amplitude: 0.5 G; modulation frequency: 100 kHz.

The generation of adjacent Cu species within G_4_-Cu^2+^_n_ (n ≥ 12) could also be supported by ESR measurements. The ESR spectrum of G_4_-Cu^2+^_2_ in CHCl_3_ exhibited a mononuclear Cu^2+^ signal around 320 mT (Figure 1). The intensity of the Cu^2+^ signal of G_4_-Cu^2+^_n_ decreased with increasing n from 2 to 12, and then the spectra of G_4_-Cu^2+^_n_ (n ≥ 12) were almost silent. It is known that the spin–spin interaction between closely related Cu^2+^ ions decreases the intensity of the Cu^2+^ signal [40], revealing the main formation of adjacent Cu^2+^ species within G_4_-Cu^2+^_n_ (n ≥ 12).

To gain insight into the coordination environment of the Cu^2+^ species of G_4_-Cu^2+^_12_, XAFS analysis was carried out [32]. The Cu K-edge X-ray absorption near edge structure (XANES) spectrum of G_4_-Cu^2+^_12_ exhibited an edge peak at 8982 eV. This peak might be ascribed to the shake-down transition involving a 1s→4p transition with simultaneous LMCT [41]. Fourier transforms (FT) of the *k*^3^-weighted extended X-ray absorption fine structure (EXAFS) spectrum of G_4_-Cu^2+^_12_ showed a scattering peak at 1.8 Å corresponding to the Cu-N and Cu-Cl shells based on comparison with those of CuCl_2_ [42] and Cu(ImH)_4_SO_4_ [43]. Curve-fitting analysis revealed that the Cu species were surrounded by two N and two Cl atoms.

**Figure 2:**
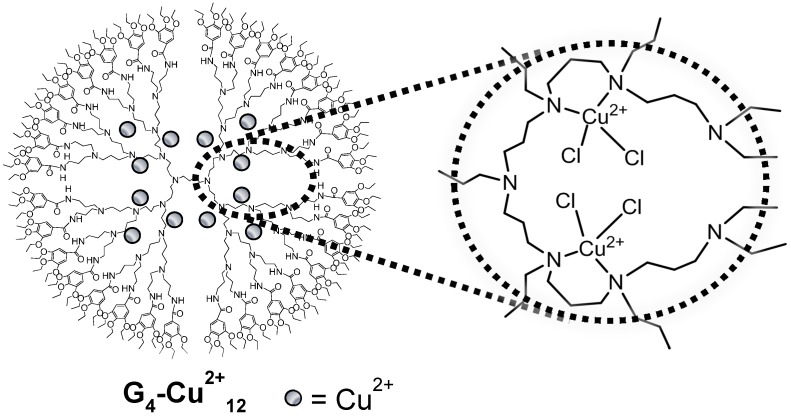
Proposed structure of Cu species within G_4_-Cu^2+^_12_.

From the combined results of UV-vis, FTIR, ESR, and XAFS analyses, a possible structure of Cu species within the nanovoids of G_4_-Cu^2+^_12_ is shown in Figure 2. CuCl_2_ species are encapsulated through the coordination to two amino groups of the branch units of G_4_-TEBA to form adjacent Cu centers.

#### 2.2.2. Magadiite-Immobilized Cu Catalyst

The XRD analysis revealed that the basal spacing of Cu^2+^-magadiite (*d*_001_ = 13.9 Å) decreased from that of the parent magadiite (*d*_001_ = 15.1 Å). The *d*_001_ value of Cu^2+^-magadiite was larger than those of proton-exchanged magadiite (H^+^-magadiite) (*d*_001_ = 11.5 Å) [44] and dehydrated magadiite (*d*_001_ = 12.8 Å) [45], confirming that Cu species were incorporated within the magadiite interlayer. The interlayer gallery of Cu^2+^-magadiite was estimated as 2.4 Å based on the subtraction of layer thickness of H^+^-magadiite [46]. The elemental analysis showed C, 1.48; H, 2.22; N, 0.60; Na, 1.49; Cu, 1.39; Si, 36.1%, suggesting that the stoichiometric amount of TMEDA to Cu^2+^ was 0.975 and Cu^2+^ species were incorporated into magadiite through a cation exchange reaction with interlayer Na^+^ cations.

The UV-vis spectrum of Cu^2+^-magadiite showed absorption bands around 690 and 280 nm corresponding to the d-d transition band and LMCT band, respectively (Figure S1) [47]. The absorbance around 690 nm indicated the presence of Cu^2+^ species coordinated by two N atoms [48]. The absorbance around 280 nm was deconvoluted into two peaks at 270 and 340 nm (Figure S2), attributed to N- and bridging OH^−^-Cu LMCT, respectively [47,48].

**Figure 3:**
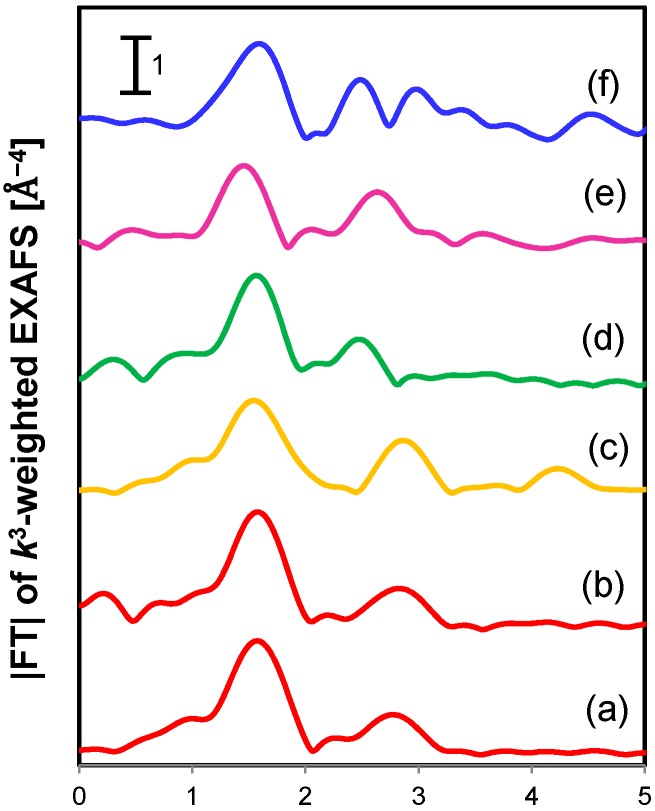
FT of *k*^3^-weighted Cu K-edge EXAFS spectra of (**a**) Cu^2+^-magadiite, (**b**) Cu^2+^-magadiite (used), (**c**) [Cu(OH)TMEDA]_2_Cl_2_, (**d**) Cu^2+^(mono)-magadiite, (**e**) CuO, and (**f**) Cu_2_O.

The Cu K-edge XANES spectrum of Cu^2+^-magadiite showed an absorption edge at 8980 eV, which was assigned to the formation of Cu^2+^ species (Figure S3) [47]. The FT of the *k*^3^-weighted EXAFS spectrum exhibited two scattering peaks around 1.5 and 2.9 Å (Figure 3). These peaks were also observed in the spectrum of a dinuclear Cu complex [Cu(OH)TMEDA]_2_Cl_2_ [39], while the scattering peaks around 2.9 Å were absent in that of the magadiite-immobilized Cu(ethylenediamine)_2_ mononuclear complex (Cu^2+^(mono)-magadiite) [49,50]. Thus, the above two peaks around 1.5 and 2.9 Å in the spectrum of Cu^2+^-magadiite were ascribed to Cu-N/O and Cu-Cu shells, respectively. The inverse FT of these peaks was well fitted to Cu-N/O and Cu-Cu shells with coordination numbers of 4.4 and 0.9, respectively (Figure 3 and Table 1). These results indicate that dinuclear Cu^2+^ species are generated and coordinated by nitrogen/oxygen ligands such as TMEDA and bridging OH^−^ groups [48].

**Table 1:** Results of curve-fitting analysis of Cu K-edge EXAFS data for Cu^2+^-magadiite ^a^.

Sample	Shell	CN ^b^	R ^c^ [Å]	σ^2 d^ [Å^2^]
[Cu(OH)TMEDA]_2_Cl_2_	Cu-O/N	4.0	2.02	-
	Cu-Cu	1.0	2.99	-
Cu^2+^-magadiite (fresh)	Cu-O/N	4.4	1.99	0.0016
	Cu-Cu	0.9	2.97	0.0042
Cu^2+^-magadiite (used)	Cu-O/N	4.5	1.98	0.0060
	Cu-Cu	1.1	2.93	0.0022

^a^ The region of 1.0–3.3 Å in FT of samples was inversely transformed. ^b^ Coordination number. ^c^ Interatomic distance. ^d^ Debye-Waller factor.

**Figure 4:**
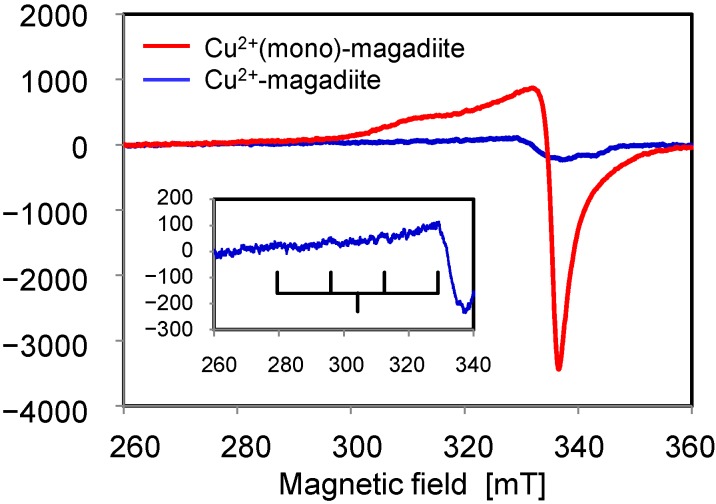
ESR spectra of magadiite-immobilized Cu^2+^ complexes recorded with the following parameters: temperature: 298 K; power: 10.0 mW; modulation amplitude: 0.5 G; modulation frequency: 100 kHz.

In ESR measurements, the intensity of the Cu^2+^ signal of Cu^2+^-magadiite was much lower than that of Cu^2+^(mono)-magadiite (Figure 4). This result was ascribed to the spin–spin interaction of closely related Cu^2+^ species [40]. The ESR spectrum of Cu^2+^-magadiite yielded a *g*-value of 2.263 and hyperfine splitting A of 165 cm^−1^. These two values indicate the presence of Cu^2+^ species in 2N2O coordination environments [51].

**Figure 5:**
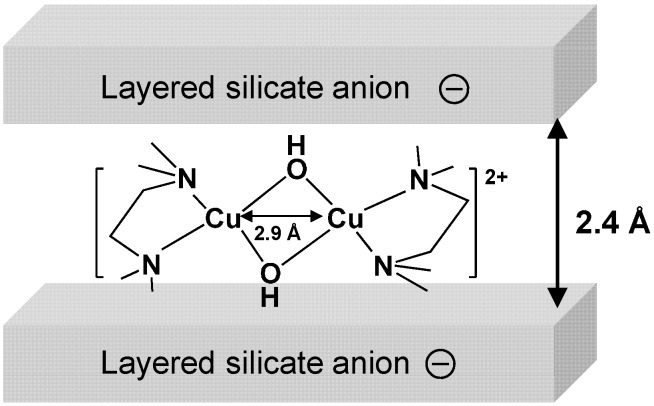
Proposed structure of Cu species in Cu^2+^-magadiite.

Consequently, we propose the structure of Cu species in Cu^2+^-magadiite (Figure 5). Adjacent Cu species (Cu···Cu ≈ 2.9 Å) coordinated by TMEDA and oxygen ligands such as bridging OH^−^ are immobilized in the interlayer nanospace of magadiite.

### 2.3. Oxidative Coupling of DMP Using Immobilized Cu Complex Catalysts

#### 2.3.1. G_4_-Cu^2+^_n_-Catalyzed Oxidative Coupling of DMP

The oxidative coupling reactions of DMP (**1a**) using various G_4_-Cu^2+^_n_ (n = 2–24) were examined at 323 K under 1 atm of O_2_ in CHCl_3_ (Table 2). The amount of encapsulated Cu^2+^ ions to G_4_-TEBA (n) strongly affected the activity and selectivity. Among the tested G_4_-Cu^2+^_n_, G_4_-Cu^2+^_12_ exhibited the highest activity and selectivity for the C–C coupling, affording the desired product DPQ (**2a**) and 3,3',5,5'-tetramethylbiphenol (TMBP, **3a**), which is known as the reaction intermediate for **2a** [19], in 55% and 10% yields, respectively, where the selectivity for the C–C coupling reached 97% (entry 3). Prolonging the reaction time allowed complete conversion of **1a** giving **2a** in 97% yield (entry 6). When decreasing n from 12 to 2, both the conversion of **1a** and the selectivity for **2a** decreased to 9% and 44%, respectively (entries 1 and 3). The increase of n from 12 to 24 diminished the conversion of **1a** to 16% while maintaining the high selectivity for the C–C coupling (entries 3 and 5).

**Table 2:** Oxidative coupling of DMP using various Cu-amine catalysts. 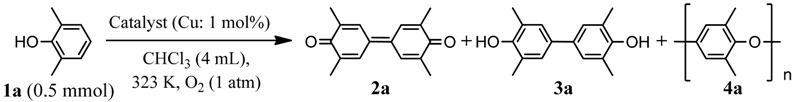

Entry	Catalyst	Solvent	Time [h]	Conv. ^a^ [%]	Sel. to	Yield ^a^ [%]		
					C-C ^b^ [%]	2a	3a	4a
1	G_4_-Cu^2+^_2_	CHCl_3_	6	9	44	4	0	4
2	G_4_-Cu^2+^_8_	CHCl_3_	6	25	68	8	9	7
3	G_4_-Cu^2+^_12_	CHCl_3_	6	67	97	55	10	2
4	G_4_-Cu^2+^_16_	CHCl_3_	6	34	88	15	15	4
5	G_4_-Cu^2+^_24_	CHCl_3_	6	16	87	7	7	2
6	G_4_-Cu^2+^_12_	CHCl_3_	18	>99	97	97	trace	2
7	G_4_-Cu^2+^_12_^c^	CHCl_3_	6	29	96	22	6	1
8	CuCl_2_-TEA	CHCl_3_	6	9	44	1	3	5
9	CuCl_2_-TMPDA	CHCl_3_	6	98	46	41	4	52
10	PEI-Cu^2+^	CHCl_3_	6	11	63	3	4	1
11	G_4_-Cu^2+^_12_	MeOH	6	97	46	32	12	47
12	G_4_-Cu^2+^_12_	CH_3_CN	6	9	33	0	3	0
13	G_4_-Cu^2+^_12_	TFT	18	>99	96	96	trace	2
14 ^d^	G_4_-Cu^2+^_12_	CHCl_3_	24	>99	97	97 ^e^	trace	2

^a^ Determined by ^1^H NMR standard technique. ^b^ Calculated from the ratio of yield of (**2a** + **3a**) to conv. of **1a**. ^c^ G_4_-Cu^2+^_12_ was treated with HCl(aq) (0.01 N, 0.25 mL) before the reaction. ^d^ Reaction conditions: G_4_-Cu^2+^_12_ (Cu: 18 µmol), **1a** (1.10 g, 9 mmol), CHCl_3_ (16 mL), 323 K, 24 h, O_2_ (5 atm). ^e^ Isolated yield.

To prove the high efficiency of G_4_-Cu^2+^_12_, several tertiary amine compounds were used instead of G_4_-TEBA [52]. The low-molecular-weight amines such as triethylamine (TEA) and *N*,*N*,*N*',*N*'-tetramethyl-1,3-propanediamine (TMPDA) could not promote the selective oxidative coupling of **1a**, resulting in the formation of PPE (**4a**) as a main product (entries 8 and 9). In addition, the use of PEI-Cu^2+^ gave only 3% yield of **2a** with 63% selectivity (entry 10), demonstrating that G_4_-Cu^2+^_12_ acts as a unique and efficient catalyst for the oxidative C–C coupling of DMP to DPQ.

The choice of solvent significantly influenced the catalytic activity and selectivity of G_4_-Cu^2+^_12_ for the oxidative coupling reaction of DMP to DPQ. Among the solvents tested, CHCl_3_ gave the highest yield of **2a** (97% yield). Although the high conversion of **1a** was achieved in MeOH, the selectivity for the C–C coupling was only 46% (entry 11). CH_3_CN was not effective, resulting in the formation of a slight amount of **3a** (entry 12). It is noteworthy that under the reaction conditions using G_4_-Cu^2+^_12_ in α,α,α-trifluorotoluene (TFT) solvent, **2a** was obtained in 96% yield (as determined by ^1^H-NMR analysis) and existed as a solid after the reaction (entry 13). Thus, **2a** was easily obtained from the reaction mixture by simple filtration. The recovered TFT solution containing G_4_-Cu^2+^_12_ was reusable with retention of its efficiency during the recycling experiments (in 93% yield) [47].

**Scheme 1:**
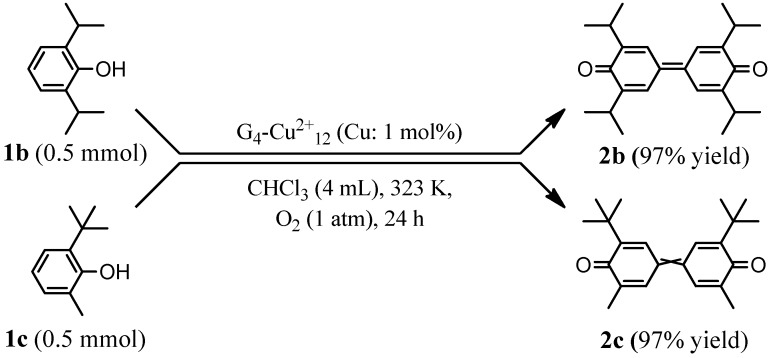
G_4_-Cu^2+^_12_-catalyzed oxidative coupling of phenol derivatives.

Under the optimized reaction conditions, the G_4_-Cu^2+^_12_-catalyzed oxidative coupling of other phenol derivatives was investigated. G_4_-Cu^2+^_12_ efficiently catalyzed the C–C selective coupling reaction of 2,6-substituted phenols including 2,6-diisopropylphenol (**1b**) and 2-*tert*-butyl-6-methylphenol (**1c**) to afford corresponding diphenoquinone derivatives (**2b** and **2c**) in 97% yields for 24 h (Scheme 1). These results differ from those obtained with *mushroom tyrosinase*, where both **1b** and **1c** were not fully converted even after extending the reaction time [16]. The coupling reaction of unsubstituted phenol or phenol derivatives with electron-withdrawing chlorine atoms did not proceed, which is similar to the results obtained by other Cu catalyst systems [16,18].

A gram-scale reaction of **1a** was carried out using G_4_-Cu^2+^_12_. 1.10 g of **1a** was selectively converted to 1.04 g (97% isolated yield) of **2a** (Table 2, entry 14) [32]. In this case, G_4_-Cu^2+^_12_ exhibited both high turnover number (TON) and turnover frequency (TOF), reaching 485 and 20.2 h^−1^, respectively. These values were much higher than those of reported catalyst systems: *mushroom* *tyroinase* (TON, 48; TOF, 5.33 h^−1^) [16] [(α,α'-bis[(*N*-methyl-2-pyridyl)ethylamino]-2-fluoro-*m*-xylene)Cu_2_(MeCN)_2_] [ClO_4_]_2_ (TON, 8.6; TOF, 1.43 h^−1^) [17], [Cu_2_(bpnp)(μ-OH)(TFA)_3_] (TON, 100; TON, 4.16 h^−1^) [18], and H_5_PV_2_Mo_10_O_40_ (TON, 40; TOF, 10 h^−1^) [19].

#### 2.3.2. Heterogeneous Oxidative Coupling Reaction of DMP Using Cu^2+^-Magadiite

Although heterogeneous catalysts are superior to homogeneous catalysts in terms of handling, separation, and reuse, a heterogeneous catalyst for the oxidative C–C coupling of DMP to DPQ has never been reported. Therefore, we focused on development of the solid support-immobilized Cu complex catalyst for the selective C–C coupling of DMP to DPQ.

The catalytic activities of a series of the Cu complexes immobilized to solid supports were investigated in the oxidative coupling of **1a**. The results are summarized in Table 3. Cu^2+^-magadiite efficiently promoted the oxidative coupling reaction under 1 atm of O_2_ at 328 K, affording a 67% yield of **2a** for 12 h (entry 1). When prolonging the reaction time to 18 h, the yield of **2a** reached 95% with the formation of a 4% yield of **4a** (entry 2). The use of Cu^2+^-SiO_2_ decreased the selectivity for **2a** from 95% to 60% (entry 6 *vs.* entry 1). Cu^2+^-mordenite exhibited low activity and selectivity to give **2a** in 27% yield with 53% selectivity (entry 7). In addition, Cu^2+^(mono)-magadiite having monomeric Cu^2+^ species gave poor yields of **2a** (entry 8).

**Table 3:** Oxidative coupling of DMP catalyzed by Cu complexes immobilized to solid supports. ^a^

Entry	Catalyst	Time [h]	Conv. ^b^ [%]	Yield ^b^ [%]	
				2a	4a
1	Cu^2+^-magadiite	12	75	67	3
2	Cu^2+^-magadiite	18	>99	95	4
3 ^c^	Cu^2+^-magadiite	18	>99	94	4
4 ^d^	Cu^2+^-magadiite	18	>99	94	4
5 ^e^	Cu^2+^-magadiite	48	>99	95	3
6	Cu^2+^-SiO_2_	12	>99	60	3
7	Cu^2+^-mordenite	12	51	27	2
8	Cu^2+^(mono)-magadiite	12	15	2	2

^a^ Reaction conditions: catalyst (Cu: 17.5 µmol), **1a** (0.4 mmol), CHCl_3_ (3.5 mL), MeOH (0.5 mL), 328 K, O_2_ (1 atm). ^b^ Determined by ^1^H NMR standard technique. ^c^ 1st reuse. ^d^ 2nd reuse. ^e^ Cu^2+^-magadiite (Cu: 0.58 µmol), **1a** (0.4 mmol), CHCl_3_ (3.5 mL), MeOH (0.1 mL), 353 K, O_2_ (10 atm).

Cu^2+^-magadiite was also applicable to the oxidative coupling reaction of other phenol derivatives having electron-donating alkyl groups such as **1b** and **1c** to afford the corresponding diphenoquinones **2b** and **2c** in 97% yields for 24 h. As in the case of G_4_-Cu^2+^_12_, the oxidative coupling of phenol or 2,6-dichlorophenol did not occur using Cu^2+^-magadiite.

**Figure 6:**
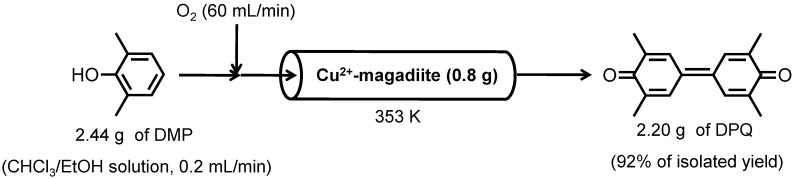
A continuous flow reaction system of Cu^2+^-magadiite-catalyzed oxidative coupling of DMP to DPQ.

One of the significant advantages of inorganic solid supports over organic polymer supports is their high durability. After the oxidative coupling reaction of **1a**, Cu^2+^-magadiite could be recovered by simple filtration and reused without any loss of activity or selectivity (Table 3, entry 2 *vs*. entries 3 and 4). Cu^2+^-magadiite catalyzed the selective coupling of **1a** even under a higher temperature of 353 K to afford **2a** in 95% yield with high TON of 655 (Table 3, entry 5). This TON value is the highest among previously reported catalysts. These results demonstrated that Cu^2+^-magadiite represents a durable heterogeneous catalyst for the oxidative coupling of DMP to DPQ. Furthermore, Cu^2+^-magadiite was applied to a continuous flow reactor system [53,54]. Cu^2+^-magadiite (0.8 g) was placed in a tubular stainless-steel reactor, and 2.44 g of DMP dissolved in CHCl_3_/EtOH (500 mL, 4/1 v/v) and O_2_ were passed through it, successfully giving 2.20 g of DPQ (92% isolated yield) (Figure 6). These experiments demonstrate that the development of heterogeneous catalysts for the selective oxidative C–C coupling of DMP offers a more efficient and practical synthetic method of DPQ from DMP.

## 3. Discussion

**Figure 7:**
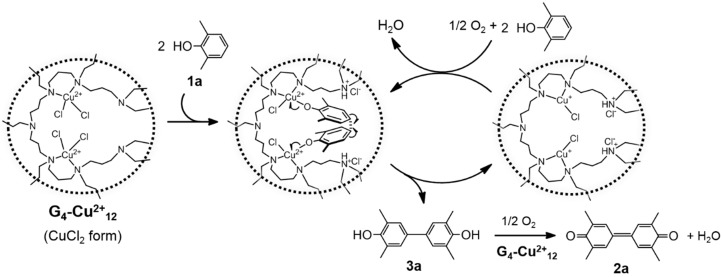
Proposed reaction mechanism of efficient oxidative coupling of DMP to DPQ using G_4_-Cu^2+^_12_.

In the case of G_4_-Cu^2+^_n_, the selectivity for the C–C coupling (Section 2.3.1., Table 2) was closely correlated with the structure of the Cu^2+^ species within G_4_-Cu^2+^_n_ (Section 2.2.1.). G_4_-Cu^2+^_n_ (n ≤ 8) having mononuclear Cu^2+^ species gave poor selectivity, whereas G_4_-Cu^2+^_n_ (n ≥ 12) having adjacent Cu^2+^ species exhibited high selectivity. These results demonstrate that the adjacent Cu^2+^ species within the dendrimer are the active species for the selective C–C coupling of DMP. The higher activity of G_4_-Cu^2+^_12_ than those of G_4_-Cu^2+^_n_ (n = 16 and 24) might be due to the presence of non-coordinated tertiary amino groups in G_4_-Cu^2+^_12_. The free amino groups in G_4_-Cu^2+^_12_ would act as base sites to promote the facile formation of Cu^2+^-phenolate species by trapping the accompanying HCl [55], thus delivering the superior activity of G_4_-Cu^2+^_12_. To investigate the accelerating effect of the free amino groups, G_4_-Cu^2+^_12_ (Cu: 5 μmol) treated with HCl(aq) (0.01 N, 0.25 mL) was used for the coupling reaction for 6 h. The conversion of **1a** resulted in only 29% (Table 2, entry 7), indicating that the basic sites of free amino groups of the dendrimer play a key role in the efficient C–C coupling reaction. The high efficiency of G_4_-Cu^2+^_12_ is ascribed to the concerted catalysis between the adjacent Cu species and the free amino groups. Furthermore, Liu *et al.* reported that a dinuclear copper phenolate species is a key intermediate for the selective C–C coupling in the oxidative coupling of DMP to DPQ [18]. From the above facts, we propose the reaction mechanism of the G_4_-Cu^2+^_12_-catalyzed oxidative C–C coupling of **1a** as shown in Figure 7.

Initially, a ligand exchange reaction between a Cl species of Cu^2+^ and **1a** occurs to give a Cu^2+^-phenolate anion species associated with the formation of HCl, which is trapped by a tertiary amino group of the dendrimer. The two Cu^2+^-phenolate anions within the nanovoids are suitably oriented for the C–C selective coupling reaction through the one-electron oxidation of the phenolate anion, giving **3a**. The two generated Cu^+^ species are oxidized by O_2_ to regenerate the Cu^2+^ species. Successive oxidation of **3a** yields the final product **2a**.

The proposed reaction mechanism is well supported by the following control experiments. *In* *situ* UV-vis analysis was carried out during the reaction of equimolar amounts of G_4_-Cu^2+^_12_ with **1a** under an Ar atmosphere for 24 h to afford **2a** and **3a** (Scheme S1) [47]. The d-d transition adsorption band derived from the Cu^2+^ species around 740 nm gradually decreased and finally disappeared at 24 h (Figure S4) [47]. These results support the one-electron transfer from **1a** or **3a** to the Cu^2+^ species, followed by the formation of the C–C coupling products. In addition, **3a** is confirmed as a reaction intermediate of **2a** by quantitative production of **2a** from **3a** [32].

The main roles of the regularly arranged tertiary amino groups of the nanovoid are (1) coordination to Cu ions to generate unique adjacent Cu species, (2) facile promotion of the ligand exchange of Cu-Cl with DMP by the base sites trapping of HCl, and (3) accumulation of both adjacent Cu species and basic sites within the confined nanovoids. The concerted catalysis between the adjacent Cu species and the base sites within G_4_-Cu^2+^_12_ enabled the efficient coupling of DMP to DPQ.

In the case of the magadiite-immobilized Cu catalysts, Cu^2+^-magadiite exhibited much higher activity and selectivity compared to Cu^2+^(mono)-magadiite (Section 2.3.2., Table 3, entries 1 and 8), showing that the active species of oxidative C–C coupling of DMP is the dinuclear Cu species generated in the layer nanospace of magadiite. Bridging OH^−^ groups coordinating the adjacent Cu species might deprotonate DMP [40,48] to promote the formation of two Cu^2+^-phenolate species, giving the C–C coupling product through a similar pathway to that in G_4_-Cu^2+^_12_. To clarify whether DMP can access the active dinuclear Cu species in the interlayer, the nanospace of magadiite was estimated by XRD analysis. When Cu^2+^-magadiite was soaked into CHCl_3_/MeOH solvent, the basal spacing of Cu^2+^-magadiite increased from 13.9 to 14.8 Å, meaning that the interlayer gallery of Cu^2+^-magadiite expanded from 2.4 to 3.2 Å. This value is larger than the minimum thickness of DMP and DPQ (molecular size of DMP: *ca*. 1.8 × 6.7 × 7.0. Å; DPQ: *ca*. 1.8 × 6.7 × 9.3. Å) [56], confirming that DMP can react with dinuclear Cu species within the interlayer nanospace and the product DPQ can be removed. As described in Section 2.3.2., Cu^2+^-magadiite maintained its high activity and selectivity during the reuse experiments (Table 3, entries 3 and 4). The Cu content in the used catalyst was the same as in the fresh one evidenced by ICP-AES analysis (Cu: 1.39 wt %). The FT of the *k*^3^-weighted EXAFS spectrum and the curve-fitting result of used Cu^2+^-magadiite were similar to those of the fresh one (Figure 3 and Table 1). These data show that the dinuclear Cu species remained unchanged after the coupling reaction. The dinuclear Cu^2+^ species in the nanospace is stabilized through the electrostatic interaction between the Cu^2+^ species and the layered silicate anion of magadiite, providing high durability of Cu^2+^-magadiite. The prominent catalysis of Cu^2+^-magadiite could be ascribed to the dinuclear Cu species, which were generated and stabilized within the two-dimensional interlayer nanospace.

## 4. Experimental Section

### 4.1. Preparation of G_4_-Cu^2+^_n_

G_4_-NH_2_ was synthesized from the NH_2_-terminated third-generation PPI dendrimers (G_3_-NH_2_) by the divergent method [57]. Surface modification of G_4_-NH_2_ with 3,4,5-triethoxybenzamide groups was carried out using a previously reported procedure to afford G_4_-TEBA [26]. The mixture of CuCl_2_ (5 µmol) and G_4_-TEBA (0.42 µmol in the case of preparation of G_4_-Cu^2+^_12_) was stirred for 2 h in 1.5 mL of CHCl_3_/CH_3_CN (2:1 v/v) under an Ar atmosphere at 298 K [29]. The resulting solution was evaporated to give G_4_-Cu^2+^_12_ as a brown, waxy solid.

### 4.2. Oxidative Coupling of DMP Using G_4_-Cu^2+^_n_

**1a** (0.5 mmol) was added to 4 mL of a CHCl_3_ solution of G_4_-Cu^2+^_n_ (Cu: 5 µmol) and the mixture was stirred under O_2_ (1 atm) at 333 K in a Schlenk flask. Following completion of the reaction, hexadecane was added as an internal standard and the mixture was treated with aqueous 6 N HCl, after which the organic phase was separated and characterized by ^1^H NMR.

### 4.3. Preparation of Cu^2+^-Magadiite 

Magadiite was synthesized by hydrothermal reaction from SiO_2_ (Wakogel Q-69), NaOH, and deionized water according to the previously reported paper [33]. Next, Cu(ClO_4_)_2_·6H_2_O (100 µmol) and TMEDA (100 µmol) were dissolved in MeOH (10 mL) at 298 K, and magadiite (0.4 g) was added into the Cu-TMEDA solution. The resulting mixture was further stirred for 6 h at 313 K. After the reaction, the obtained solid was filtered, washed with MeOH (100 mL), and dried to afford a light blue powder.

### 4.4. Oxidative Coupling of DMP Using Cu^2+^-Magadiite

**1a** (0.5 mmol) and Cu^2+^-magadiite (0.08 g, Cu: 17.5 µmol) were placed in a Schlenk flask, and then 4 mL of CHCl_3_/MeOH (7:1 v/v) solvent was added. The reaction mixture was heated at 328 K for appropriate times under O_2_ (1 atm). After the reaction, hexadecane was added as an internal standard and the mixture was filtered and subjected to ^1^H NMR analysis.

### 4.5. Cu^2+^-Magadiite-Catalyzed Oxidative Coupling of DMP Using Continuous Flow Reactor

A stainless steel column (inner diameter: 5 mm; length: 150 mm) was filled with Cu^2+^-magadiite (0.8 g) and SiO_2_ (Wakogel C-400HG, 0.15 g). A solution of **1a** in CHCl_3_/EtOH (2.44 g: 20 mmol in 500 mL of CHCl_3_/EtOH (v/v 4:1), 0.2 mL/min) and O_2_ (60 mL/min) were passed through the column at 353 K for 42 h. After the reaction was completed, the solvent was evaporated. The obtained supernatant was passed through a silica gel column (Wakogel C-200). Byproducts were first removed by elution with hexane/ethyl acetate (v/v = 3/2) and **2a** was subsequently eluted with hexane/CH_2_Cl_2_ (v/v = 1/3). The evaporation of the solvent gave **2a** in 92% yield (2.20 g).

## 5. Conclusions

We developed efficient Cu complex catalysts by using the nanospaces of PPI dendrimers or magadiite for the selective oxidative C–C coupling of DMP to DPQ. The PPI dendrimer accommodated Cu ions into the internal nanovoids to form adjacent Cu species, which promoted the highly efficient regioselective coupling reaction. We also devised an immobilized dinuclear Cu complex catalyst by incorporation of Cu complexes into the interlayer nanospace of magadiite, and successfully demonstrated the selective oxidative coupling reaction of DMP to DPQ. This is the first example of a selective heterogeneous catalyst for the oxidative C–C coupling of DMP to DPQ. The magadiite-immobilized Cu catalyst has significant advantages such as (1) recoverability of catalyst, (2) catalyst reusability, (3) high durability, and (4) applicability to a flow reactor system, providing the highly practical and green synthesis of DPQ from DMP and O_2_. These strong catalytic performances of PPI dendrimer-encapsulated and magadiite-immobilized Cu complex catalysts for the selective oxidative C–C coupling of DMP to DPQ are ascribed to the adjacent Cu species generated within the nanospaces of the PPI dendrimer and magadiite. We believe that the regulated nanospaces of structurally ordered materials would serve as a platform for preparation of multinuclear metal species to provide strong catalytic performance.

## Supplementary Materials

Supplementary materials can be accessed at: http://www.mdpi.com/1420-3049/20/02/3089/s1.
