# Supplementary material for: Selective C–C Coupling Reaction of Dimethylphenol to Tetramethyldiphenoquinone Using Molecular Oxygen Catalyzed by Cu Complexes Immobilized in Nanospaces of Structurally-Ordered Materials
Source: Molecules. 2015 Feb 12;20(2):3089–106. doi: 10.3390/molecules20023089 (PMC6272262; doi:10.3390/molecules20023089)
Supplement: Supplementary file 1 [file molecules-20-03089-s001.pdf]

Supplementary Materials

General

Third-generation (G_3, where the subscript denotes the generation) poly(propylene imine) (PPI) dendrimer (G_3-NH_2) was purchased from SyMO-Chem B.V. (Eindhoven, The Netherlands). Polyethyleneimine 10000 (Mw = 10,000) was purchased from the Junsei Chemical Co., Ltd (Tokyo, Japan). All other chemicals were purchased from Wako Pure Chemicals (Tokyo, Japan), the Tokyo Kasei Co. (Tokyo, Japan), and Sigma-Aldrich Inc. (St. Louis, MO, USA) and used after appropriate purification. ^{1}H NMR and ^{13}C NMR spectra were obtained using a JEOL GSX-270 or JNM-ESC400 spectrometer and chemical shifts (δ) were reported in ppm downfield from tetramethylsilane. UV-vis spectra were obtained using a JASCO V-660 (Tokyo, Japan) and Fourier transformed infrared spectra (FTIR) were acquired on a JASCO FTIR-4100 spectrometer (Tokyo, Japan). Electron spin resonance (ESR) spectra were recorded at the Xband using a Bruker EMX-10/12 spectrometer (Kanagawa, Japan). Cu K-edge X-ray absorption data were collected in the quick mode and recorded in the transmission mode at the BL14B2 and BL01B1 stations attached to the Si(311) monochromator at SPring-8 (JASRI), Harima, Japan (Proposal Nos. 2012B1869, 2013B1414, 2014A1513, 2014B1235). The data analysis was carried out using the REX2000 program ver. 2.5.7 (Rigaku, Tokyo, Japan). Powder X-ray diffraction (XRD) patterns were recorded using a Philips X'Pert-MPD (Eindhoven, The Netherlands) with Cu-Kα radiation. Elemental analysis was carried out using CHN corder MT-5 (Yanako, Tokyo, Japan) and inductively coupled plasma-atomic emission spectroscopy (ICP-AES) measurements were performed using ICPS-8100 (Shimadzu, Kyoto, Japan). The continuous flow reactor (column oven and stainless steel column) was purchased from YMC Co., Ltd. (Kyoto, Japan).

Preparation of Cu^{2+}(mono)-Magadiite

Cu^{2+}(mono)-magadiite was prepared in the same manner as reported by Kim *et al.* [1]. A Cu mononuclear complex Cu(ethylenediamine)$_2$(ClO_4)$_2$ was synthesized by addition of ethylenediamine (1.3 mL, 20 mmol) to the MeOH solution containing $Cu(ClO_4)_2 \cdot 6H_2O$ (10 mmol), and the resulting solution was refluxed for 4 h. After the reaction, the solution was cooled to room temperature to precipitate the purple solid. The precipitate was filtered and washed with ethanol to afford Cu(ethylenediamine)$_2$(ClO_4)$_2$. Next, Cu(ethylenediamine)$_2$(ClO_4)$_2$ (100 μmol) was dissolved in EtOH (10 mL), and magadiite (0.4 g) was added into the above solution. The resulting mixture was further stirred for 6 h at 313 K. After the reaction, the obtained solid was filtered, washed with EtOH (100 mL), and dried to afford a light purple powder.

Reuse Experiment of G_4-Cu^{2+}_{12}

1a (0.5 mmol) was reacted using fresh G_4-Cu^{2+}_{12} (Cu: 5 μmol) in 4 mL α,α,α-trifluorotoluene (TFT), after which the synthesized **2a** was removed from the reaction mixture by filtration. **1a** (0.5 mmol) was added to the filtered TFT solution, still containing the original G_4-Cu^{2+}_{12}, and the mixture was vigorously stirred at 323 K for 18 h. By repeating this process, it was determined that the G_4-Cu^{2+}_{12} catalyst could be reused without significant loss of its activity and selectivity; the yield of **2a** from the first reaction, as determined by ^{1}H NMR, was 96%, while the isolated yields were 83% (fresh catalyst), and 93% (first reuse).

ESR Measurement

The spectra were recorded at the Xband using a Bruker EMX-10/12 spectrometer with a 100 kHz magnetic field modulation at a microwave power level of 10.0 mW. G_4-Cu^{2+}_n (n = 2, 8, 12, 16, and 24; Cu: 0.5 μmol) in $CHCl_3$ (0.1 mL) was placed in a quartz ESR tube under an Ar atmosphere, and measured at 298 K. In the case of the heterogeneous Cu catalysts, the Cu catalyst (Cu: 0.5 μmol) was introduced in a quartz ESR tube, evacuated at room temperature, and subjected to analysis at 298 K.

XAFS Measurement

The Cu K-edge XAFS spectrum of G_4-Cu^{2+}_{12} was recorded in transmission mode at room temperature. Fourier transforms of k^3-weighted EXAFS spectra were performed in the 4 Å < k < 12 Å range to obtain radial structural functions. Curve-fitting analysis was performed with the inverse FT of the 1.2 Å < R < 2.4 Å range. The coordination numbers (CN) and interatomic distances (R) were estimated by curve-fitting analysis using Cu-Cl and Cu-N shell parameters obtained from reference samples of $CuCl_2$ [2] and $Cu(ImH)_4SO_4$ [3], respectively. In the case of Cu^{2+}-magadiite, the spectrum was recorded at 10 K using a cryostat. Curve-fitting analysis was conducted with the inverse FT of the 1.0 Å < R < 3.3 Å range, and the CN and R were estimated by curve-fitting analysis using Cu-N/O and Cu-Cu shell parameters obtained from a reference sample of $[Cu(OH)TMEDA]_2Cl_2$ [4].

Product Identification

The reaction products were identified by ^{1}H and ^{13}C NMR and, in each case, the chemical shifts of the products were in agreement with those reported in the literature, as summarized below.

3,3',5,5'-Tetramethyldiphenoquinone (DPQ, **2a**) (CAS-RN 4906-22-3) [5]

3,3',5,5'-Tetramethylbiphenyl-2,2'-diol (TMBP, **3a**) (CAS-RN 2417-04-1) [5]

Poly(2,6-dimethylphenylene ether) (PPE, **4a**) (CAS-RN 42397-25-1) [6]

3,3',5,5'-Tetraisopropyldiphenoquinone (**2b**) (CAS-RN 2178-51-0) [5]

3,3'-Di-*tert*-butyl-5,5'-dimethyldiphenoquinone (**2c**) (CAS-RN 2417-00-1) [5] (Likely a mixture of cis and trans isomers [7]).

Scheme and Figures

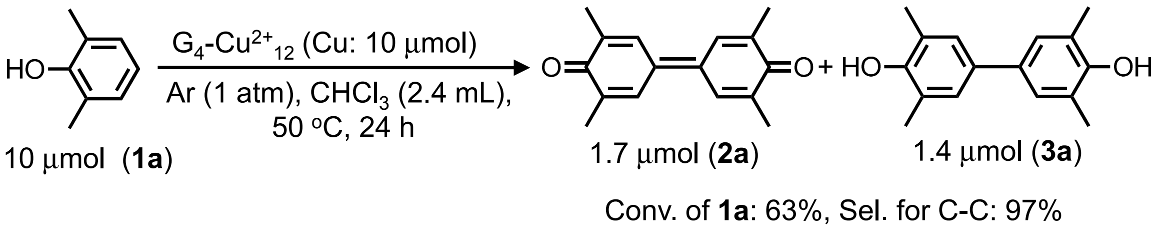

Scheme S1. G_4-Cu^{2+}_{12}-catalyzed oxidative coupling of DMP under an Ar atmosphere.

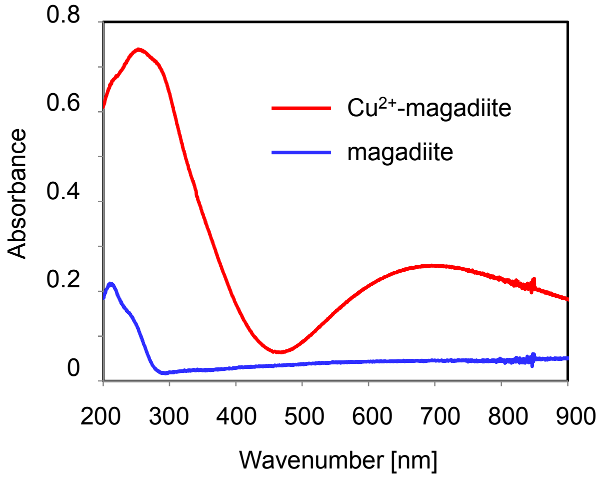

Figure S1. UV spectra of Cu^{2+}-magadiite and magadiite.

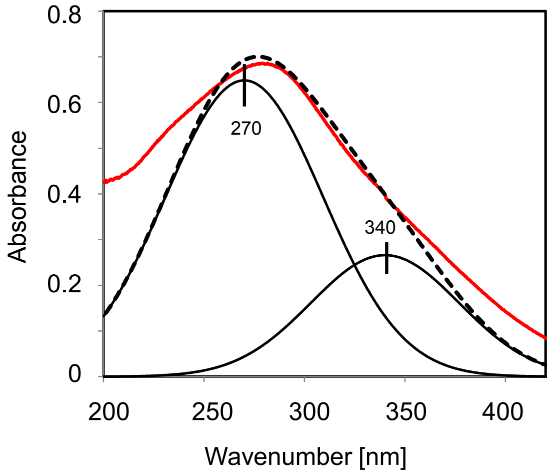

Figure S2. UV-vis spectrum of LMCT band of Cu^{2+}-magadiite.

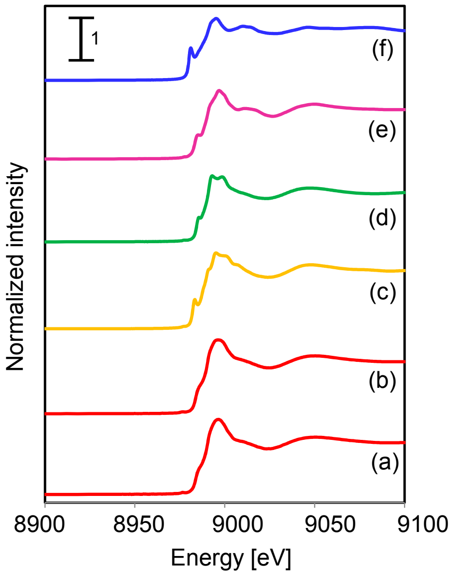

Figure S3. Cu K-edge XANES spectra of (a) Cu^{2+}-magadiite, (b) Cu^{2+}-magadiite (used), (c) $[Cu(OH)TMEDA]_2Cl_2$, (d) Cu^{2+}(mono)-magadiite, (e) CuO, and (f) Cu_2O.

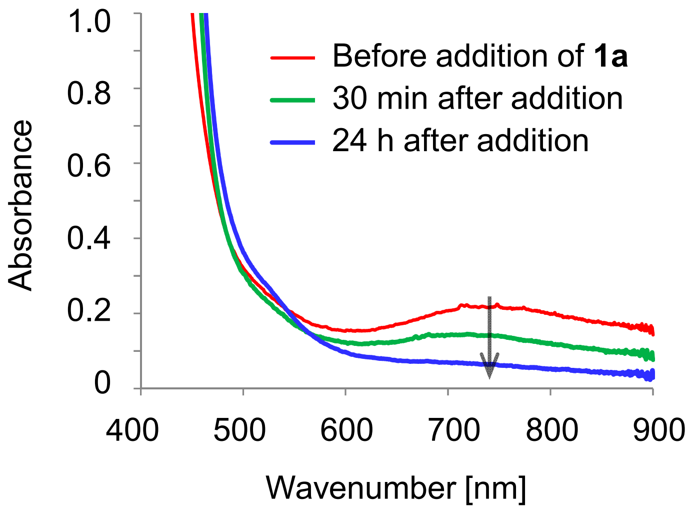

Figure S4. Simultaneous *in situ* UV-vis spectra recorded during G_4-$Cu^{2+}{}_{12}$-catalyzed oxidative coupling of DMP under an Ar atmosphere as shown in Scheme S1.

References

1. Choy, J.-H.; Kim, D.-K.; Park, J.-C.; Choi, S.-N.; Kim, Y.-J. Intracrystalline and electronic structure of copper(II) complexes stabilized in two-dimensional aliminosilicate. *Inorg. Chem.* **1997**, *36*, 189–195.
2. Wells, A.F. 333. The crystal structure of anhydrous cupric chloride, and the stereochemistry of the cupric atom. *J. Chem. Soc.* **1947**, 1670–1675.
3. Fransson, G.; Lundberg, B.K.S. Metal complexes with mixed ligands. 4. The crystal structure of tetrakisimidazole Cu(II) sulphate, $Cu(C_3H_4N_2)_4SO_4$. *Acta Chem. Scand.* **1972**, *26*, 3969–3976.
4. Tromp, M.; van Strijdonck, G.P.F.; van Berkel, S.S.; van den Hoogenband, A.; Feiters, M.C.; de Bruin, B.; Fiddy, S.G.; van der Eerden, A.M.J.; van Bokhoven, J.A.; van Leeuwen, P.W.N.M.; *et al.* Multitechnique approach to reveal the mechanism of copper(II)-catalyzed arylation reactions. *Organometallics* **2010**, *29*, 3085–3097.
5. Liao, B.-S.; Liu, Y.-H.; Peng, S.-M.; Liu, S.-T. Efficient oxidative coupling of 2,6-disubstituted phenol catalyzed by a dicopper(II) complex. *Dalton Trans.* **2012**, *41*, 1158–1164.
6. Gui, L.; Wang, H.; Shentu, B.; Weng, Z. Synthesis and characterization of low-molecular-weight poly(2,6-dimethyl-1,4-phenylene oxide) in water. *J. Appl. Poly. Sci.* **2013**, *128*, 2919–2926.
7. Rieker, A.; Kessler, H. Notiz zur rotationsisomerie bei p-diphenochinonen. *Chem. Ber.* **1969**, *102*, 2147–2149.
